# Writers and readers of H3K9me2 form distinct protein networks during the cell cycle that include candidates for H3K9 mimicry

**DOI:** 10.1042/BSR20231093

**Published:** 2023-10-27

**Authors:** Gareth Pollin, Thiago M. De Assuncao, Salomao Doria Jorge, Young-In Chi, M. Cristine Charlesworth, Benjamin Madden, Juan Iovanna, Michael T. Zimmermann, Raul Urrutia, Gwen Lomberk

**Affiliations:** 1 Linda T. and John A. Mellowes Center for Genomic Sciences and Precision Medicine, Medical College of Wisconsin, Milwaukee, WI, U.S.A.; 2 Division of Research, Department of Surgery, Medical College of Wisconsin, Milwaukee, WI Center, Medical College of Wisconsin, Milwaukee, WI, U.S.A.; 3 Medical Genome Facility, Proteomics Core, Mayo Clinic, Rochester, MN, U.S.A.; 4 Centre de Recherche en Cancérologie de Marseille (CRCM), INSERM U1068, CNRS UMR 7258, Aix-Marseille Université and Institut Paoli-Calmettes, Parc Scientifique et Technologique de Luminy, Marseille, France; 5 Department of Biochemistry, Medical College of Wisconsin, Milwaukee, WI, U.S.A.; 6 Clinical and Translational Sciences Institute, Medical College of Wisconsin, Milwaukee, WI, U.S.A.; 7 Department of Pharmacology and Toxicology, Medical College of Wisconsin, Milwaukee, WI, U.S.A.

**Keywords:** cell cycle, chromatin, epigenetics, mass spectrometry, molecular dynamics, protein-protein interactions

## Abstract

Histone H3 lysine 9 methylation (H3K9me), which is written by the Euchromatic Histone Lysine Methyltransferases EHMT1 and EHMT2 and read by the heterochromatin protein 1 (HP1) chromobox (CBX) protein family, is dysregulated in many types of cancers. Approaches to inhibit regulators of this pathway are currently being evaluated for therapeutic purposes. Thus, knowledge of the complexes supporting the function of these writers and readers during the process of cell proliferation is critical for our understanding of their role in carcinogenesis. Here, we immunopurified each of these proteins and used mass spectrometry to define their associated non-histone proteins, individually and at two different phases of the cell cycle, namely G1/S and G2/M. Our findings identify novel binding proteins for these writers and readers, as well as corroborate known interactors, to show the formation of distinct protein complex networks in a cell cycle phase-specific manner. Furthermore, there is an organizational switch between cell cycle phases for interactions among specific writer–reader pairs. Through a multi-tiered bioinformatics-based approach, we reveal that many interacting proteins exhibit histone mimicry, based on an H3K9-like linear motif. Gene ontology analyses, pathway enrichment, and network reconstruction inferred that these comprehensive EHMT and CBX-associated interacting protein networks participate in various functions, including transcription, DNA repair, splicing, and membrane disassembly. Combined, our data reveals novel complexes that provide insight into key functions of cell cycle-associated epigenomic processes that are highly relevant for better understanding these chromatin-modifying proteins during cell cycle and carcinogenesis.

## Introduction

Emerging evidence implicates repressive H3K9 methylation as an important transcriptional regulatory and epigenetic pathway in tumorigenesis [1]. H3K9 methylation is driven by at least 6 distinct SET domain-containing enzymes; namely SUV39H1 and SUV39H2, which establish H3K9 trimethylation (H3K9me3) at constitutive heterochromatin, SETDB1 and SETDB2, involved in silencing of retrotransposons, interspersed repetitive elements, and centromere-associated repeats, as well as EHMT1 (GLP) and EHMT2 (G9a), which are predominantly responsible for gene repression via H3K9 mono- and di-methylation (H3K9me1 and H3K9me2) at euchromatic loci [2]. EHMT1 and EHMT2 form homo- and heterodimeric complexes [3]. The heterodimeric EHMT1/EHMT2 complex is considered the predominant functional H3K9me2 KMT due to its essentiality for mouse embryonic life and the inability of one to compensate for loss of the other [3,4]. However, subsequent studies found that these two enzymes display different tissue-specific expression profiles and have distinct roles in certain tissues, suggesting some independent functions [5,6]. For instance, studies from our laboratories and others have demonstrated that EHMT2 is dispensable for proper pancreas development in mice [7]. The HP1 sub-family of CBX chromodomains are also readers of H3K9me2 and H3K9me3, binding the mark through their N-terminal chromodomain [8–10]. Notably, both writers and readers of the H3K9me pathway are associated with predominantly tumor promotion in various cancers by modulating diverse processes, such as cell cycle regulation [11,12], although the underlying molecular mechanisms remain to be further elucidated.

During the cell cycle, a series of exquisitely coordinated events occur, which include DNA replication, histone synthesis, and duplication of chromatin structure during S phase and chromosome condensation and segregation for mitotic cell division during M phase. To ensure genomic and epigenomic integrity along with proper progression of the cell cycle, chromatin modifications and their regulators are an integral part of this process by regulating appropriate gene expression and chromatin structure [13]. Furthermore, programs for exiting the cell cycle, such as cellular quiescence or senescence, require distinct gene expression networks and accompanying chromatin organization [14]. EHMT2 levels oscillate through the cell cycle with peaks of protein levels in S and M phase, and this protein has been found to localize at replication forks, interacting with PCNA and DNMT1 [15,16]. HP1 proteins, CBX3 and CBX5, are extensively post-translationally modified throughout the cell cycle with an increase in phosphorylation during mitosis [17]. We have previously reported that phosphorylation of CBX3 by Aurora A in G2/M localizes this subpopulation to the spindle pole and is necessary for proper cell division [18]. A similar phosphorylation site on CBX5 localizes this protein to the centromere-kinetochore complex and plays a role in maintaining mitotic fidelity [19]. A more recent study has demonstrated that CBX5 mechanically stabilizes both interphase and mitotic chromosomes to maintain nuclear shape and chromosomal stability [20]. While these H3K9 methylation writers and readers have emerged as critical determinants of cell cycle progression and cell fate decisions, there is limited knowledge on the composition of the complexes and how their interactions fluctuate during the cycle cell.

In the present study, we used a multi-tiered approach to better define the interactomes of H3K9me writers (EHMT1 and EHMT2) and readers (CBX3 and CBX5), during the G1/S and G2/M cell cycle phases. Immunopurified complexes were identified by mass spectrometry, and these interacting protein networks were validated using several data science-based algorithms. Notably, our results reveal known and novel non-histone-related interactions, along with distinct cell cycle-specific protein networks. Given that EHMT1 and EHMT2 also methylate non-histone proteins, we analyzed the interacting proteins for the presence of an H3K9-like recognition domain, defining a list of novel candidate targets for histone mimicry. Combined, the present study extends the current paradigm on pathways that link, directly or indirectly, to these epigenetic regulators and offers unique insight into cell cycle-associated epigenomic processes that are highly relevant to human cancer.

## Materials and methods

### Tissue culture and cell synchronization

Following our prior publication, HeLa cells were used to obtain G1/S and G2/M lysates after double thymidine synchronization and release [18]. Briefly, HeLa cells were obtained from the American Type Culture Collection (ATCC) and maintained according to the manufacturer’s protocol. For synchronization, thymidine (Sigma-Aldrich) was added at a concentration of 2 mM to asynchronous cells for 18 h. Cells were released for 9 h in complete growth media followed by a second 2 mM thymidine block for 17 h. Subsequently, cells were released from the thymidine block in complete growth media for 2 or 9 h, corresponding to cells in S phase or mitosis, respectively, as we have shown [18].

### Immunoprecipitation (IP) and Liquid Chromatography Electrospray Ionization Tandem Mass Spectrometry (LC-MS/MS) analysis

Antibodies to EHMT1 (Abcam, ab41969), EHMT2 (Thermo Scientific, PA5-34971), CBX3 (Abcam, ab10480), CBX5 (Abcam, ab77256) or control IgG (Abcam, ab37415) were conjugated through disuccinimidyl suberate (DSS) cross-linking to Protein A/G Magnetic Beads (Thermo Fisher Scientific). Centrifuged pellets of cells in S phase or mitosis were lysed with IP Lysis/Wash Buffer (Thermo Fisher Scientific), and lysates were incubated with antibody conjugates overnight at 4°C. Immunoprecipitated complexes were washed and eluted to resolve on 4–15% Criterion™ Tris-HCl Protein Gels (Bio-Rad). Gels were subsequently visualized with BioSafe™ Coomassie Stain (Bio-Rad). Each gel lane was divided into eight sections, destained in 50% acetonitrile/50 mM Tris pH 8.1. Proteins were reduced with the addition of 50 mM TCEP/50 mM Tris pH 8.1 at 55°C for 30 min. Next, the samples were alkylated by adding 25 mM iodoacetamide/50 mM Tris pH 8.1 and incubated in the dark for 30 min at room temperature. In-gel digestion was carried out at 37°C overnight with 0.15 µg trypsin (Promega Corporation, Madison WI) in 25 mM Tris pH 8.1/0.0002% Zwittergent 3-16, followed by peptide extraction with 2% trifluoroacetic acid and acetonitrile. Extracts were pooled and subsequently used for LC/ESI-MS/MS analysis at the Mayo Clinic Proteomics Core. The pooled extracts were concentrated, and the proteins were identified by nano-flow liquid chromatography-electrospray tandem mass spectrometry (nanoLC-ESI-MS/MS) using a Thermo Scientific Q-Exactive Plus Mass Spectrometer (Thermo Fisher Scientific) coupled to a Thermo Ultimate 3000 RSLCnano HPLC system. The extracts were then loaded onto a 250 nl OPTI-PAK trap (Optimize Technologies) custom packed with Michrom Magic C18 solid phase (Michrom Bioresources). Chromatography conditions were: solvent A water; 0.2% formic acid; 2% acetonitrile and solvent B water; 80% acetonitrile; 10% isopropanol. The Q-Exactive Plus mass spectrometer set up was a FT full scan from 340 to 1600 *m*/*z* at resolution 70,000 (at 200 *m*/*z*), followed by HCD MS/MS scans on the top 15 ions at resolution 17,500. The MS1 AGC target is set to 1e6 and the MS2 target is set to 1e5 with max ion inject times of 50 and 100 ms, respectively. Only ions with charge states of +2, +3, and +4 were selected for MS2 fragmentation and dynamic exclusion placed the selected ions on an exclusion list for 30 s. Tandem mass spectra were extracted by msconvert version 3.0.9134. All MS/MS samples are analyzed using Mascot (Matrix Science; version 2.4.0) and X! Tandem (The GPM, thegpm.org; version X! Tandem Sledgehammer (2013.09.01.1)). Mascot and X! Tandem were set up to search a combined Swissprot human database with reverse decoy (40620 entries), assuming the digestion enzyme strict trypsin and with a fragment ion mass tolerance of 0.020 Da and a parent ion tolerance of 10.0 PPM. Glu->pyro-Glu of the n-terminus, ammonia-loss of the n-terminus, gln->pyro-Glu of the n-terminus, and oxidation of methionine were specified in X! Tandem as variable modifications and carbamidomethyl of cysteine was specified as a fixed modification. Oxidation of methionine and carbamidomethyl of cysteine were specified in Mascot as variable modifications. Scaffold (version Scaffold_4.8.3, Proteome Software Inc.) was used to validate MS/MS based peptide and protein identifications. Peptide identifications were accepted if they were established at greater than 95.0% probability by the Scaffold Local FDR algorithm and contained at least two identified peptides. The decoy false discovery rate was less than 1%. Protein probabilities were assigned by the Protein Prophet algorithm [21]. Proteins that contained similar peptides and could not be differentiated based on MS/MS analysis alone were grouped to satisfy the principles of parsimony. Protein comparisons were made with ratios of total peptide counts against the IgG negative control.

### Bioinformatics analysis

Prior to data processing, proteins that contained less than four peptides were considered low enrichment proteins and removed from downstream analysis. Next, peptide counts which failed to reach a log2 foldchange threshold in the bait sample versus the IgG control sample were removed as potential contaminants to ensure that hits were specific to bait-protein interactions rather than non-specific binding [22]. As a final step to remove background and contaminant from non-specific binding, the CRAPome contaminant lists for HeLa cells highlighted actin, keratin, tubulin, and histone proteins as common contaminants out of 30 control experiments [23]. To ensure downstream gene ontologies were not distorted by potential contaminants, actin, keratin, histone and tubulin proteins were removed from the potential hits. All genes that failed to pass this rigorous screening process were classed as False hits, while the proteins that did pass were and termed as True hits and thus potential interactor candidates (Supplementary Data). To highlight the increased specificity from the rigorous filtration, we found a substantial increase in the ratios of IgG total peptide counts compared with our bait samples. For example, after filtration, we found a 4-fold increase for the EHMT1 bait protein over the IgG in the G1/S dataset, highlighting the value of this filtration method, and similar was observed with other bait proteins. The bait specific potential candidates were collated into a master proteomic matrix for further analysis. The nuclear interactome was filtered using the Gene Ontology Cellular Component term nucleus (GO:0005634) to define the nuclear proteins, according to AmiGO 2 version: 2.5.17. Heatmaps were generated through the ComplexHeatmap package on R [24], and Venn diagrams were generated with the web tool https://bioinformatics.psb.ugent.be/webtools/Venn/. Transcriptome microarray data for the HeLa cell synchronized cells was obtained from the Gene Expression Omnibus (GEO) database under accession number GSE27031. The data was accessed using the GEOquery package on R studio [25]. Differential gene expression analysis was performed using the limma package in R [26]. Nuclear interactors which were not present in the transcriptome dataset were excluded from all downstream analysis. All data processing and statistical analyses were performed using R version 4.1.1.

### Protein-protein interaction network

Protein interactions from the LC/ESI-MS/MS experiments fitting the present study’s criteria for novel histone mimicry were searched against the STRING database version 11.5 for protein–protein physical interactions [27]. Edges were included if their confidence score was high (>0.7), and the interaction network was visualized in Cytoscape (v3.9.1) [28]. CORUM database (Release July 2017) was used to obtain protein complexes that were connecting 171 proteins that were candidates for histone H3K9 mimicry [29]. Interaction connectivity networks were generated with proteins in the interactomes of the four baits and using combined data from BioPlex [30], CCSB [31], and HPRD [32] reference interactome sets.

### Enrichment-based clustering

Biological processes GO enrichment analysis was carried out using the clusterProfiler and enrichGO R package. The interactions were annotated using org.Hs.eg.db with a *P*-value cutoff = 0.05. Gene ontology for biological processes was visualized by heatmaps using ggplot2 [33].

### Identification of potential non-histone targets

Ten peptide sequences based on known EHMT2 lysine methylation targets (ERCC6 K448, HDAC1 K432, ERCC6 K170, DNMT1 K70, KLF12 K313, ACIN1 K654, ERCC6 K297, EHMT2 K185, EHMT1 K205, ERRC K1054, CDYL K13, Histone H3K9, and WIZ K1162) were aligned through Clustal Omega [34] and entered through MEME (Multiple Em for Motif Elicitation) to discover recurring, fixed-length patterns [35]. The pattern was then completed using the substrate analysis from Rathert et al., 2008. The sequence was shortened to a 4-residue motif [GCSAKLN] [R] [K] [TGQSVMALNKER] which was used as the sequence pattern through Scansite 4.0 [36]. Scansite revealed over 17,000 proteins containing the motif. We then used the rationale that if a protein contained the proposed motif and interacted with EHMT1 and EHMT2 that the lysine methylation mark could be established. Subsequently, a protein was only identified as a candidate if it also interacted with either CBX3 or CBX5, suggesting that the established mark was being ‘read’. Further evidence was also provided by phosphosite to detect previous identification of lysine methylation at our proposed motif [37]. All lists can be found in Supplementary Data S1.

### EHMT2-substrate peptide complex structure modeling

High-resolution (1.7 Å) crystal structure of a histone H3K9M mutant peptide (1–11)-bound form of human EHMT2 SET domain (PDB access code: 5T0K) was used in our study. This structure contains the natural cofactor S-adenosylmethionine (SAM) and the structural zinc ions. The H3K9M mutant peptide was replaced with the natural H3K9me1 peptide from PDB access code 3HNA and used as the WT peptide. For non-histone substrate complex modeling, amino acid substitutions were made within the Discovery Studio suite version 19.1 (Dassault Systèmes BIOVIA) by mutating the corresponding residues of the H3K9me1 peptide and selecting the side chain rotamer causing the least steric hindrance with the surrounding residues. These structures were subjected to the same energy minimization and molecular dynamics (MD) simulation protocol as the EHMT2 SET domain-histone H3K9me1 peptide complex.

### H3K9me1 peptide pharmacophore model

The energy minimized EHMT2 SET domain-histone H3K9me1 peptide complex was used to develop the pharmacophore model in Discovery Studio. The co-complexed peptide was extracted to create the model from the bioactive conformation. The maximum number of features considered was 6 within the minimum distance of 2.5 Å between features in the generated pharmacophore. The protocol also added excluded volumes (EVs; size = 1.2 Å) to indicate regions of inaccessibility by the bioactive conformation; however, these were concealed from view for better visualization of the model. The pharmacophore model with the highest selectivity score as predicted by a Genetic Function Approximation (GFA) model was selected [38].

### Molecular dynamics (MD) simulations and time-dependent interaction energy calculations

MD simulations were performed using the CHARMm36 all-atom-force-field [39] implemented in the Discovery Studio with a 2 fs time step. Molecular models were simulated in a simplified distance-dependent implicit solvent environment at a dielectric constant of 80 and pH 7.4, and no further parameterization of a non-standard residue (K9me1), SAM cofactor, and zinc ions. Energy minimization commenced for 5,000 steps using steepest decent followed by 5,000 steps of conjugate gradient to relax the protein structure that was obtained under the stressed crystal environment. Each system of histone H3 peptide- and non-histone substrate peptide-bound models was independently heated to 300 K over 200 ps and equilibrated for 500 ps followed by 10 ns production simulation under NPT ensemble by changing the initial seed (100 ns total). Structures during unconstrained dynamics simulation were recorded every 10 ps to give a total of 1000 frames for analyses. Molecular interaction free energies were calculated using the protocol implemented in the Discovery Studio. Non-bonded interactions were monitored and dynamic interaction energies (van der Waals and electrostatic energies) for each of 10 replicates were calculated from the MD trajectories using the CHARMm36 force field and the implicit distance-dependent dielectric solvent model and averaged.

## Results

### The cell cycle defines cellular and nuclear interacting protein networks linked by H3K9me writers and readers

**Figure 1:**
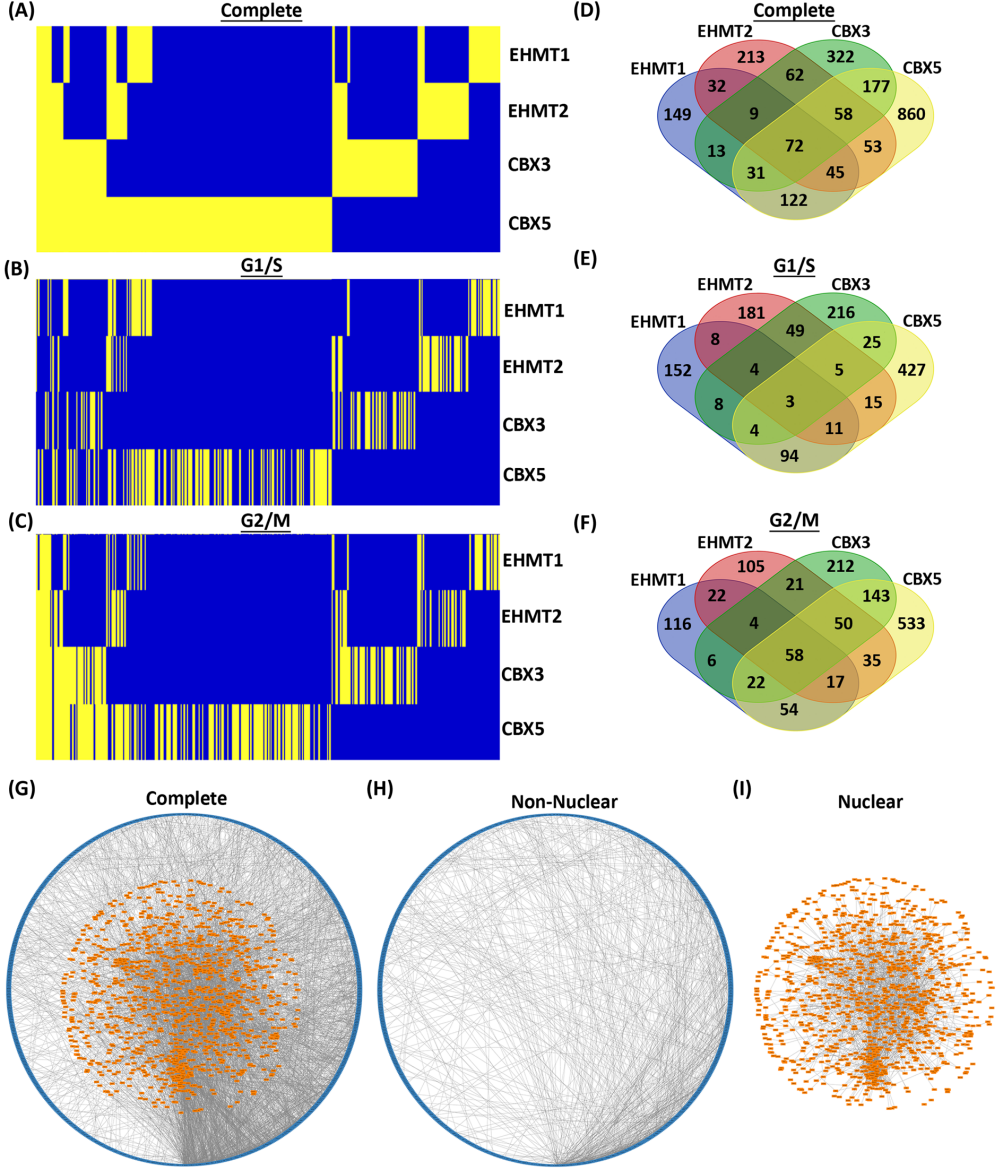
Complete interactomes of H3K9me writers and readers as defined by cell cycle phase Heatmaps are shown for the cellular interactomes of the epigenomic writers, EHMT1 and EHMT2, and readers, CBX3 and CBX5, as a complete set (**A**) and then separated by interactions occurring in G1/S (**B**) and G2/M (**C**). Yellow is a positive hit for protein–protein interaction while blue represents no interaction. Venn diagrams exhibit the number of unique and common cellular interactors that immunoprecipitated with these epigenomic regulators in total (**D**), in G1/S phase (**E**) and in G2/M (**F**). (**G**) Complete physical interaction network between non-nuclear and nuclear proteins is driven in large part by a subset of nuclear proteins displayed toward the bottom of the network. Each node represents a protein from the EHMT–CBX interactomes with cytoplasmic, non-nuclear nodes as blue and nuclear nodes as orange. Non-nuclear genes are ordered around the circle by number of protein interactions. Sparse connectivity is noted in the non-nuclear fraction (**H**) with an average of 2.9 connections per node compared to that of the nuclear fraction (**I**) with an average of 7.0 connections.

The current study was designed to identify cell cycle specific protein networks for both H3K9me writers (EHMT1 and EHMT2) and readers (CBX3 and CBX5). For this purpose, we immunopurified each of these four proteins from HeLa cells that were synchronized in G1/S and G2/M and performed mass spectrometry analyses, as previously described [18,19], and in alignment with investigations on the interactomes of other epigenetic regulators [40–46]. We first confirmed the presence of the EHMT/WIZ/ZNF644 complex in the EHMT1 and EHMT2 affinity purifications, and similarly, for the CBX3 and CBX5 proteins, we found known complex members such as CHAMP1 (ZNF828) and CHD4 [47–50]. Interestingly, while interactions between the EHMT/WIZ/ZNF644 complex and CBX3 or CBX5 were not found during G1/S, this changed during G2/M when substantial interactions were obtained when CBX3/CBX5 were used as the bait (Supplementary File). These investigations revealed that the largest interactome linked 1418 proteins to CBX5, followed by CBX3 with 744 and then both writers, EHMT1 and EHMT2, with 473 and 544, respectively (Figure 1A). From these interactions, we found that EHMT1 maintained 110/473 (23.2%) associated proteins across G1/S and G2/M, whereas 174/473 (36.8%) were unique to G1/S, and 189/473 (40%) to G2/M (Figure 1B,C). Interestingly, interactions with EHMT2 appeared to be more dynamic with only 44/544 (8.1%) of the interactions remaining consistent across the cell cycle, compared with 232/544 (42.6%) and 268/544 (49.3%) found to be unique to G1/S and G2/M, respectively. For the readers, CBX3 interacted with 86/744 (11.6%) of the same proteins across both G1/S and G2/M, and CBX5 maintained 78/1418 (5.5%) of its interactions. Moreover, G2/M displayed the largest fraction of unique interactors for both readers (Figure 1B,C). Exclusive interactions for CBX3 were substantial with 430/744 (57.8%) of proteins associating to this reader in G2/M and 228/744 (30.6%) in G1/S. Similarly, CBX5 bound 834/1418 (58.8%) specifically in G2/M and 506/1418 (35.7%) only in G1/S. In summary, most interactions captured for each of these four epigenomic regulator proteins were phase-specific, supporting the idea that their functions fluctuate with the cell cycle.

Combined, the H3K9me writers and readers co-immunoprecipitated a total of 2218 unique proteins. From these interactions, we found that 674/2218 (30.39%) were shared by at least 2 of these epigenomic regulators (Figure 1D). Within this Cellular Interactome, we identified 1202 proteins in G1/S and 1398 in G2/M, respectively. The G1/S interactome contained 226 (18.80%) proteins shared between 2 or more epigenomic regulators (Figure 1E). Notably, EHMT1 and CBX5, accounted for 49.6% of the overlap, followed by EHMT2 and CBX3 with 27%. In comparison, the G2/M interactome revealed a higher proportion of overlap with 432 (30.90%) proteins immunoprecipitated by 2 or more epigenomic regulators (Figure 1F). Different from G1/S, CBX3 and CBX5 demonstrated a predominant overlay at 63.2%, followed by EHMT2 and CBX5 with 37%. Moreover, 382 proteins were present in both, G1/S and G2/M, of which, 60 were observed with one epigenomic regulator during G1/S and then interacted with a different one in G2/M. In summary, our H3K9me writer–reader Cellular Interactome uncovered subsets of shared interactions, which were more evident in G2/M, suggesting a selected convergence of functions during this phase of the cell cycle.

**Figure 2:**
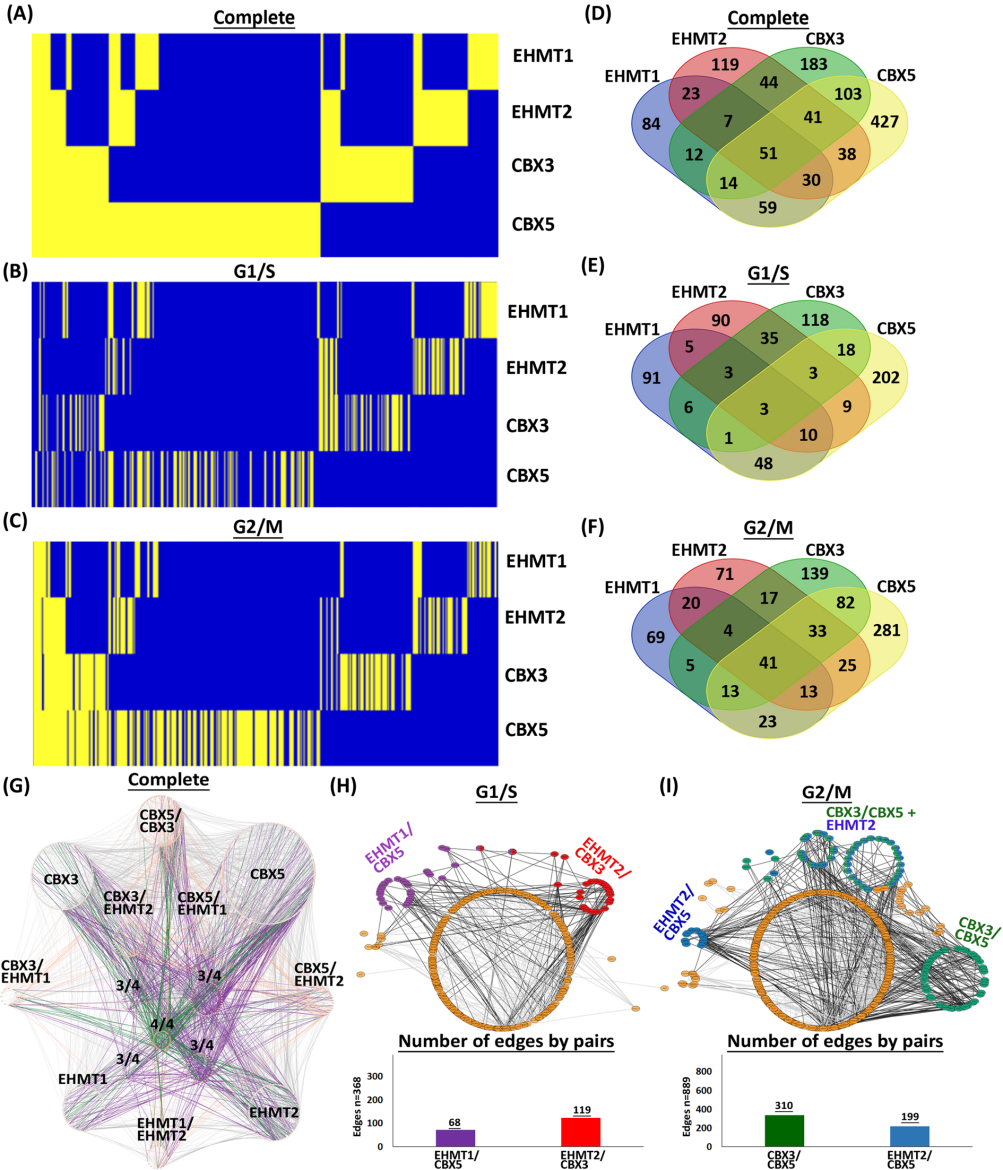
The nuclear interactomes of H3K9me writers and readers reveal robust connectivity driven by CBX readers Heatmaps display the nuclear EHMT–CBX interactomes combined (**A**) and separated into G1/S (**B**) and G2/M (**C**) interactomes. Yellow represents the presence of a protein–protein interaction, while blue indicates no interaction. Venn diagrams demonstrate the number of shared and exclusive hits from the complete nuclear interactome (**D**), as well as separated by interactions occurring in G1/S (**E**) and G2/M (**F**). (**G**) Physical interactions within the nuclear interactome based on bait protein reveal that many of the inter-set interactions are due to a few proteins in each set and that overall, the interactomes of the CBX readers, in particular CBX5, have greater interconnectivity than the EHMT writer interactomes. Edges with connections to 4 out of the 4 (4/4) bait proteins are colored green; 3/4 are purple; 2/4 are orange, and all others are gray. Physical interaction networks highlight prominent overlapping pairs formed during (**H**) G1/S and (**I**) G2/M (top panels). Bar graphs show number of edges plotted by pairs (bottom panels).

We also found that the complete H3K9me writer–reader cellular interactome comprised 1282 (58%) nuclear and 935 (42%) non-nuclear interactors. Importantly, the group of nuclear interacting proteins formed more known interconnections than the non-nuclear group (Figure 1G–I). In fact, a subset of 27 nuclear proteins (2.4%) were responsible for 31.1% of the connections to the non-nuclear proteins. Thus, this Nuclear Interactome became the focus of our subsequent analyses. Consequently, we cross-referenced our data with publicly available transcriptome microarray analysis performed on HeLa cells that were also synchronized to G1/S and G2/M phases using the double thymidine block and release [51]. We found that 96% of our interactome mapped to the transcriptome in both cell cycle phases. Thus, we extrapolated that the proteins found in our cell-cycle-specific interactomes were largely independent of expression (Supplementary Table S1). To increase stringency, we removed the 4% (55) proteins that had no expression by microarray. The now validated nuclear interactome did not affect the overall quantitative distribution of interactions observed in the complete interactome from Figure 1 (Figure 2A–C). For example, EHMT1 had the most interactions constant across G1/S and G2/M, while CBX5 was the most distinct. Furthermore, the G2/M interactome for each epigenomic regulator was more extensive than G1/S. We found that 422 of the 1228 (34.36%) nuclear proteins immunoprecipitated with 2 or more epigenomic regulators (Figure 2D). Separation by cell-cycle phase showed 642 interactions in G1/S and 836 in G2/M (Figure 2E,F). Within G1/S, 141 (21.9%) interactors were shared among 2 or more epigenomic regulators, and G2/M resulted in 276 (33%). There were 250 proteins identified in both the G1/S and G2/M datasets. Network analysis revealed that the 42 immunoprecipitated proteins in common with all four epigenomic regulators formed almost one-quarter of the nuclear interactome connections, most of which were via the CBX readers rather than EHMTs (Figure 2G). Separating the analysis by G1/S (Figure 2H) and G2/M (Figure 2I) further highlighted the difference between cell cycle phases and revealed distinct patterns of overlap. In G1/S, EHMT1 and CBX5 encompassed almost 60% of the overlap and interactor nodes connected to 68/368 edges, while EHMT2 and CBX3, which had only 27% of the overlap, connected to 119/368 (Figure 2H). Thus, while the EHMT1 and EHMT2 writers do not directly interact with the CBX3 and CBX5 readers in G1/S, remarkably, these regulatory systems organize as two distinct reader–writer pairs, namely EHMT1–CBX5 and EHMT2–CBX3, through their shared interactomes. We found that interactions with both EHMT1 and CBX5 consisted of proteins associated with DNA damage repair pathways, such as ATR, ATRIP, MTA1, BCLAF1 and others [52,53], while interactions with EHMT2 and CBX3 included proteins found at the replication fork, specifically the DNA-methylation components DNMT1, UHRF1, and USP7 [54] (Supplementary Table S2). During G2/M, the readers CBX3 and CBX5 had the largest overlap of interactors, followed by EHMT2/CBX5. In addition, all four proteins form a quantitatively larger overlapping interaction network compared with G1/S, and members of the EHMT writer complex (EHMT1, EHMT2, WIZ, and ZNF644) are part of the CBX3 and CBX5 G2/M interactomes. Unlike G1/S in which most of the overlap was observed distinctly across reader–writer pairs, the G2/M overlap is largely captured by CBX5. This finding suggests a predominant role of CBX5 among these four H3K9me epigenetic regulators during G2/M. Together, the readers involved 310/889 edges, while the EHMT2/CBX5 pair consisted of 199/889 (Figure 2I). In summary, these analyses suggest that distinct core partners within the EHMT and CBX interactomes drive most of the shared interactions with a set of common peripheral proteins during G1/S, while G2/M is dominated by the CBX3–CBX5 reader pair interactions. Thus, the overlay of our nuclear interactomes supports a greater scaffolding role for CBX readers compared to the EHMT writers.

### Cell-cycle progression links EHMT writers and CBX readers to protein complexes from different chromatin pathways

**Figure 3:**
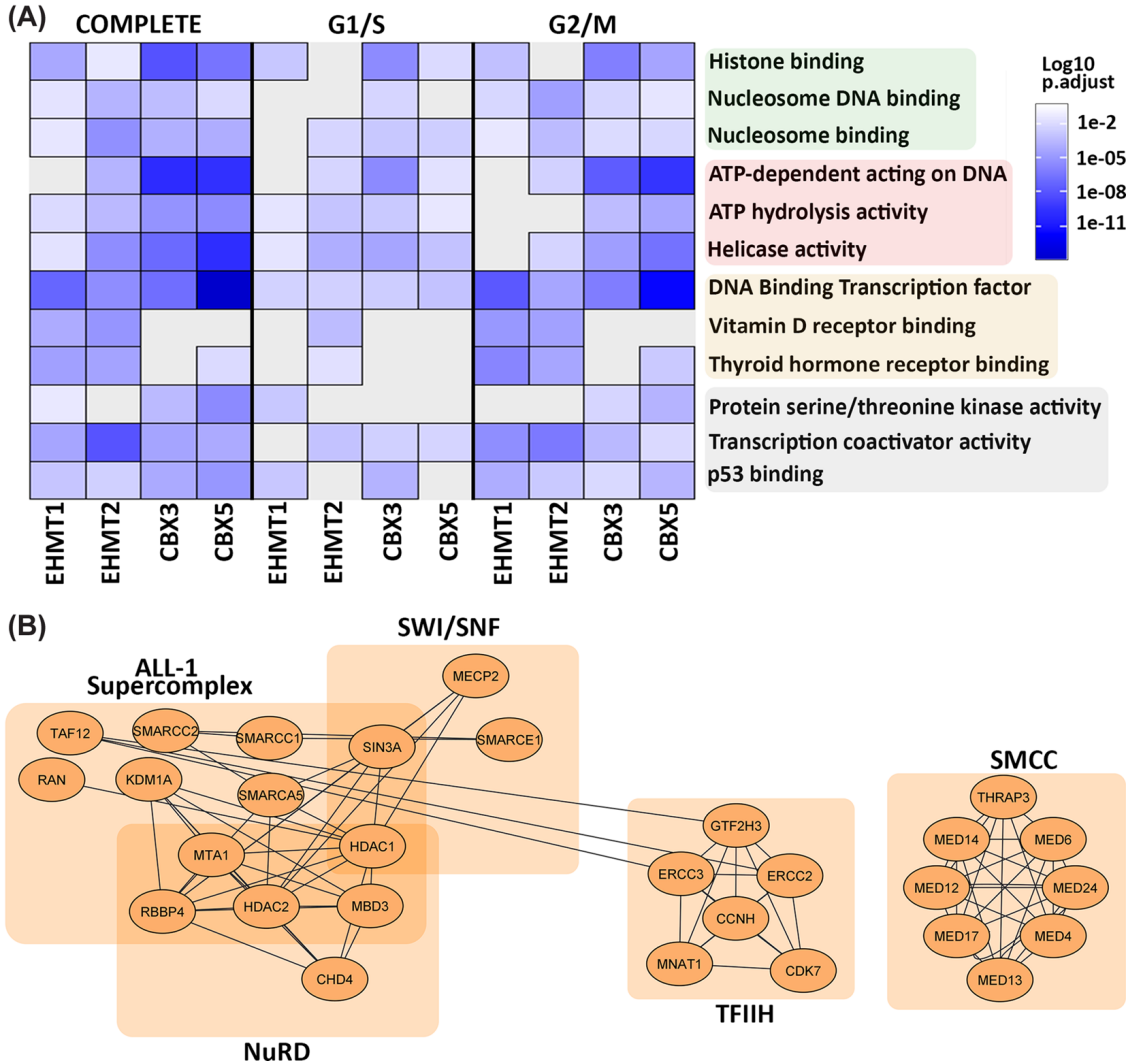
Gene ontology illustrates distinct and common molecular functions of H3K9me writers and readers by cell cycle phase (**A**) Molecular function enrichment analysis of the complete nuclear interactome and separated by G1/S and G2/M is shown to demonstrate shared and unique enrichments among EHMT writers and CBX readers depending on cell cycle phase. Color scale is depicted on the right with significance of enrichment in shades of blue and white, with grey representing no significant enrichment (n.s.). Gene Ontology terms were grouped based on shared parent terms: shaded green box indicates Chromatin binding; red for ATP-dependent activity; orange for Transcription factor binding; and gray is used for others with no shared terms. (**B**) CORUM enrichment of interactors demonstrates common core complexes emerging from the combined interactomes that are present throughout the cell cycle.

Ontological analyses of the four Nuclear Interactomes demonstrated distinct and overlapping functional enrichments during the G1/S and G2/M phases (Figure 3A). The complete nuclear datasets of the H3K9me writers and readers revealed similar ontology of child terms, which were grouped as chromatin-associated protein binding, ATP-dependent activity, and transcription factor binding. We identified additional terms that included kinase activity, transcription coactivator activity and p53 binding. Specifically, we found that during G1/S, EHMT1 did not exhibit enrichment for transcription coactivator activity, while EHMT2 or CBX5 did not show enrichment for p53 binding during this cell cycle phase. While kinase activity enrichments were identified for EHMT1 in G1/S, this functional assignment shifted exclusively to CBX3 and CBX5 in G2/M (Figure 3A, gray terms). The grouped ontologies of chromatin-associated protein binding and ATPase activity were more consistent across all four epigenomic regulators with subtle changes among child terms with the exception of EHMT1, which had no enrichment during G2/M for the latter (Figure 3A, green and red terms, respectively). Finally, enrichments for the child term DNA-binding transcription factor binding were identified across all interactomes. During the G1/S phase, EHMT2 demonstrated an exclusive association with vitamin D receptor binding and thyroid hormone factor receptor binding. However, in G2/M, both EHMT2 and EHMT1 exhibited enrichment for these ontologies, while CBX5 also displayed enrichment specifically for thyroid hormone receptor binding (Figure 3A, orange terms).

Based on the themes that emerged from the above molecular function ontologies, we investigated whether the interactors of these writers and readers form specific multi-protein complexes (Figure 3B). This information is important since inhibitors to some members of this pathway may affect other linked complexes, therefore generating side effects to a drug. We found that the ALL-1 Supercomplex (ALL-1), specifically the NuRD components (nucleosome remodeling and deacetylation), are present in both transcriptional factor and chromatin-associated protein binding terms. In addition, these terms contained components of the SMCC (SRB- and MED-containing cofactor complex) and SWI/SNF (SWItch/Sucrose Non-Fermentable) complexes. Complexes that display mechanochemical ATPase activity included TFIIH (Transcription factor IIH), which is critical for transcriptional initiation by RNA polymerase II and transcription-coupled nucleotide excision repair [55,56]. Consequently, this evidence suggests that these H3K9me writer and reader proteins are implicated in transcriptional regulation through both chromatin-associated protein binding complexes, as well as transcription factor binding. Together, this data extends our mechanistic understanding of the repertoire of functions linked to the EHMT and CBX proteins outside the canonical H3K9me pathway. Moreover, we find that the association of these H3K9me readers and writers with other enzymatic complexes, such as TFIIH and SWI/SNF, has implications for broader effects when using pharmacological agents against the latter [57–60]. Hence, this knowledge should be considered in the future when developing or testing small molecules targeting any of these pathways.

### Histone mimicry extends the repertoire of candidates for EHMT1/EHMT2-mediated methylation

**Figure 4:**
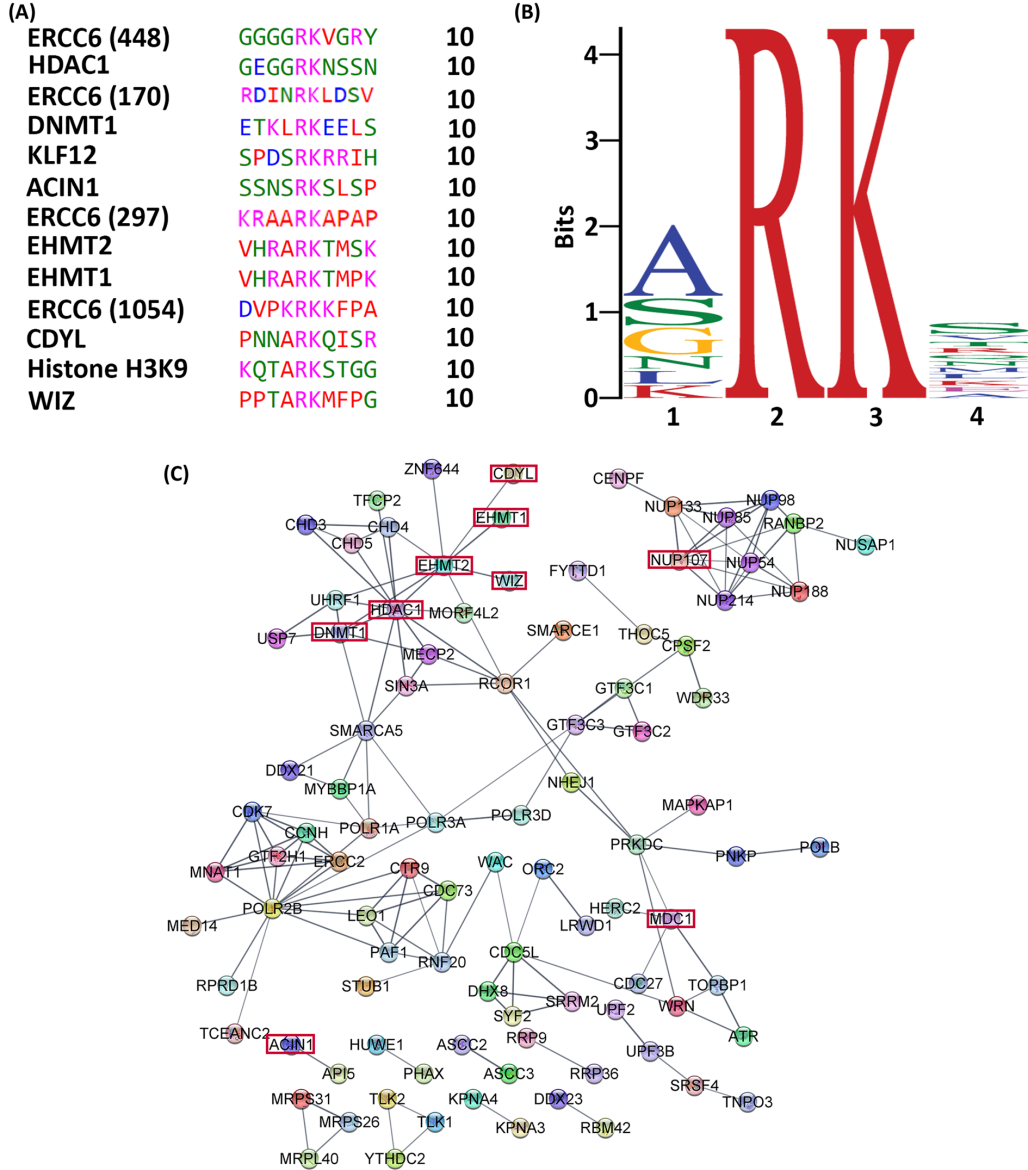
Novel candidates of histone mimicry are found in the EHMT–CBX interactomes (**A**) Sequences of previously described targeted peptides/proteins of non-histone methylation by EHMT2 were aligned using EMBL-EBI for visual representation, and (**B**) MEME was used for pattern recognition to discover novel motifs with small sample correction applied to the motif. (**C**) Physical subnetwork interactions containing 75 nodes of potential candidates of histone mimicry from our experimental data are shown. Edges indicate proteins are part of a physical complex, and edge thickness reflects the strength of data supporting the protein-protein interactions, as derived from experiments, databases, and/or predictions. Isolated nodes were removed; the minimum interaction score was set to high confidence of 0.700.

Similar to kinases, methylases recognize distinct Short Linear Motifs (SLiM), which are composed of short sequences recognized by other proteins for mediating protein binding and posttranslational modifications. For instance, the ARKS sequence within histones was the first SLiM identified to undergo targeting by EHMT1 and EHMT2 for methylation [61]. However, these writers methylate lysine residues within non-histone proteins, including WIZ, LIG1, P53, and C/EBP through a mechanism known as histone mimicry [62–65]. Herein, we derived a multi-tiered approach to identify potential histone-mimetic candidates within our interactomes. We first derived a SLiM pattern from sequence segments of 10 amino acids from known non-histone targets of EHMT2 (Figure 4A,B) [62,66]. This analysis revealed a core four-residue consensus with RK in the 2 and 3 positions, as a consistent pattern among all included peptides. Position 4 showed the largest variability, suggesting that the residue following the target lysine may not influence binding. Nevertheless, previous studies have shown that modification of this residue, such as phosphorylation, interferes with writing and/or reading the lysine [10,62]. The final SLiM used hereafter, [ASGNLKEC] [R] [K] [SVTRQNMLKEAGR], uncovered 8230 proteins within the known human proteome. We compared the nuclear interactome of EHMT1 and EHMT2, defined above, to these 8230 proteins, which revealed 313 as candidate non-histone substrates. We subsequently examined the reader interactomes for the presence of these 313 proteins, assuming that lysine methylation could be read by CBX3 and/or CBX5, consistent with previous data [67–69]. Noteworthy, we found that of the 313 candidates for histone mimicry target from our writer dataset, 54% (171) of them were also present in our reader dataset (Supplementary Table S3). Henceforth, we refer to this group of proteins as the histone mimetic dataset (Supplementary Table S4). From these 171 proteins, 65 (38.0%) were present in both, G1/S and G2/M, while 61 (35.7%) were exclusive to G2/M and 45 (26.3%) to G1/S. We utilized experimentally derived large-scale mass spectrometry data to further substantiate the methylation of these targets by EHMT1 or EHMT2 [37]. This type of analysis provided evidence to uncover 5 novel proteins and to confirm 6 proteins with K-methylation at their predicted SLiMs. Combined, these studies describe a distinct subgroup of proteins from our interactome that possess a H3K9-mimetic SLiM for EHMT1 or EHMT2.

Consequently, we investigated whether these H3K9-mimetic candidates cluster into distinct multiprotein complexes. First, among the 171 H3K9-mimetic proteins identified in both G1/S and G2/M, 75 form physical connections at high confidence (Figure 4C). Second, proteins that are known histone mimics clustered together with other candidates and within a DNA methylation predominant, repressive complex [62,70,71]. Identification of mediators and polymerase sub-units, previously implicated in EHMT2 transcriptional activation, suggests an additional layer of interaction through histone mimicry [72]. Third, we performed an analysis based on experimentally verified mammalian protein complexes [29] that contain at least 4 proteins, which uncovered 93 H3K9-mimetic candidates within 8 distinct complexes (Supplementary Figure S1). These complexes included the ALL-1 supercomplex components SWI/SNF, NuRD, and Sin3A (SIN3 transcription regulator homolog A), and the TFIIH complex that were highlighted in our ontology analyses (Figure 3B). Other significant (*P*≤0.05) complexes were CtBP, PAF1, Nup107-160, and the Spliceosome. In summary, the multi-tiered analyses applied here to our interactomes expand the repertoire of non-histone candidates that can be regulated by enzymes initially believed to only function as histone methylases.

### Molecular modeling and molecular dynamics simulations provide evidence for high-affinity interactions between EHMT2 and its histone-mimetic candidate targets

**Figure 5:**
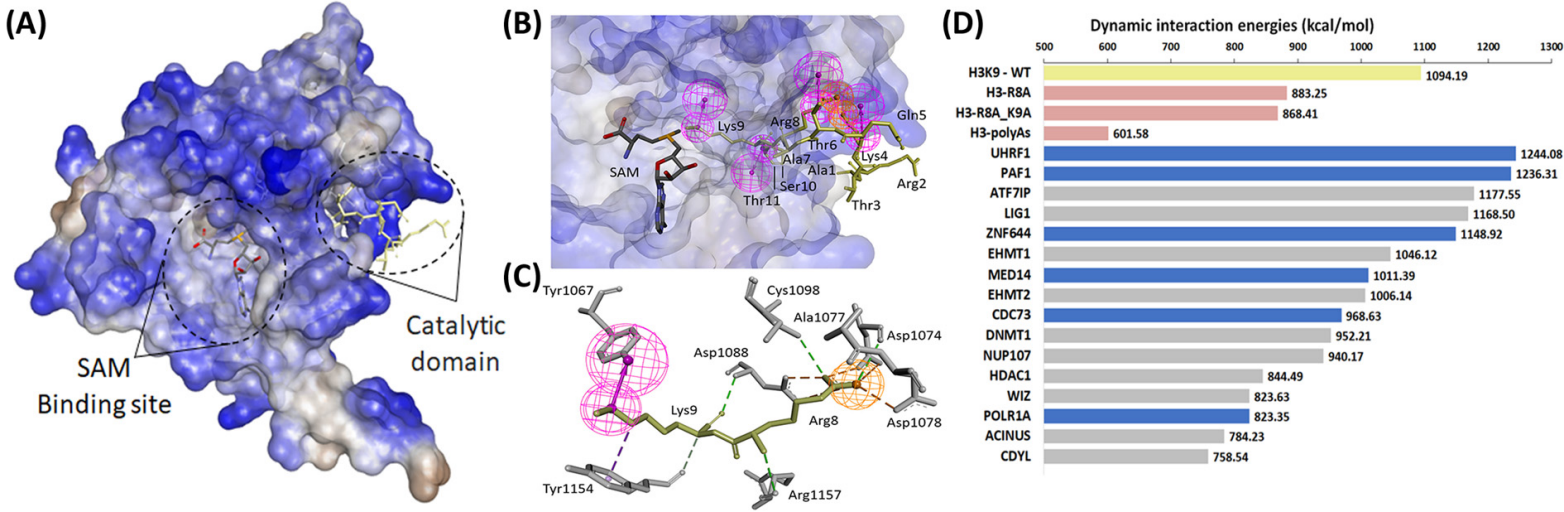
Molecular modeling of EHMT2 indicates essential residues required for correct orientation of the H3K9 peptide and molecular dynamic simulations provide evidence for high affinity interactions novel histone-mimetics (**A**) Crystal structure of the EHMT2 SET domain is shown in complex with the histone H3K9peptide. SAM binding site and KMT catalytic domain are displayed as black dashed circles. Surface representation model of EHMT2 SET domain is colored blue for hydrophilic surfaces, brownish gray for hydrophobic ones and light gray for residues in between. The H3 peptide is shown as a stick model in the catalytic domain (pale yellow), and the co-factor SAM is also represented as a stick model (carbon atoms are in gray color, oxygen in red, nitrogen in blue, sulfur in yellow) in the SAM binding site. (**B**) The EHMT2 SET domain, H3 peptide, and SAM cofactor are presented as a receptor–ligand complex pharmacophore model. The pharmacophore model is composed of four HBD features (vectored pink spheres) and two ionizable positive features (orange spheres). H3 peptide residues are labeled. (**C**) Pharmacophore features of the substrate ‘RK’ motif in stick model and key EHMT2 residues for critical interactions (dashed lines). Hydrogen bond interactions are shown in green, salt bridges in orange, and hydrophobic interactions in purple. The protein residues are show as stick in gray. (**D**) Time-dependent dynamic energy calculations of target substrate peptides for binding in the EHMT2 catalytic domain. The positive control wild-type histone H3 peptide is represented by a pale-yellow bar, and negative controls with mutated H3 residues, H3R8A, H3R8A_K9A and H3-A^10^, are represented as pink bars. Previously identified non-histone substrates (gray bars) are also shown for comparison with the novel ones discovered in the current study (blue bars). These are averaged values from 10 replicates of MD simulation data.

We used molecular modeling and dynamics simulations to further gain insight into the ability of H3K9 methylases to recognize the above-identified non-histone candidates. Fortunately, high-resolution crystal structures of the H3K9 methyltransferase SET family, including EHMT1 and EHMT2, are available, offering insight into the structural mechanism underlying substrate recognition of the H3 tail [2]. Utilizing this knowledge, we evaluated the potential recognition of a subset of H3K9-mimetic candidates. First, we generated a pharmacophore model utilizing the high-resolution crystal structure of the EHMT2 SET domain in complex with a histone H3K9me1 peptide (PDB code: 5T0K). We identified principal chemical features involved in this receptor–ligand complex. The lysine (K9) substrate occupies the hydrophobic channel that crosses the core of the EHMT2 catalytic domain and links to the co-factor SAM (S-adenosyl-methionine) binding site (Figure 5A). Our pharmacophore model identified four hydrogen bond donor (HBD) and two positive ionizable (PI) features (Figure 5B). The HBD interaction between the Nε-lysine (H3 peptide) and Y1067 of EHMT2 appears to be critical for the catalytic function of transferring the methyl group from SAM to the substrate (Figure 5C). Additionally, hydrophobic interactions with Y1154 in the EHMT2 SET domain facilitate the best alignment of K9 with the methyl group of SAM for the methyl transfer (Figure 5C) [73]. The H3R8 residue adjacent to K9 plays an important role to anchor the lysine in the binding channel, consisting of a PI feature in NH_2_^+^-R8, along with important strong hydrogen bond interactions with residues D1074, C1098, and R1157 in the entrance of the channel, as well as salt bridges with D1078 and D1088 (Figure 5C). Therefore, these core features in the interaction between the EHMT2 SET domain and the H3 tail distinguish the essential role of the ‘RK’ motif during recognition for substrate methylation.

We next performed simulations to measure predicted interaction energies. For this purpose, we developed models of EHMT2 in complex with candidate peptides and completed dynamics simulations to determine the time-dependent binding energies. Sequences of 10 amino acids from 7 novel histone mimicry candidates, with the target lysine at position 5, were selected. The candidates were chosen based on their known interactions with EHMT2, including both, repressors (UHRF1 and ZNF644) and co-activators (MED14, PLOR1A, CDC73, and PAF1) [72,74–78]. To compare the changes in the interaction energies, we modeled three negative controls: (1) the key interacting R8 residue mutated to alanine (H3-R8A), (2) the RK motif mutated to alanine (H3-R8A_K9A), or (3) the entire peptide mutated to alanine (H3-A^10^) (Figure 5D). Our results show a substantial change in the interaction energy when the R8 residue is modified to alanine (H3K9me1-WT compared with H3-R8A). The RK mutation lowered the energy in comparison to the first negative control, further demonstrating the essential role of the R8 residue. We also observed the influence of side chain-mediated interactions outside the RK motif, with the polyalanine peptide control causing additional loss of interaction energy (H3-A^10^). By comparing the effect of our controls, we can reinforce the critical role of the ‘RK’ motif during interaction with EHMT2, responsible for loss of 46% of interaction energy when compared with the H3-A^10^ (Figure 5D). The energies of the 7 novel peptide substrates were compared with the energies of the histone H3 peptide and 10 previously identified substrates for EHMT2-mediated methylation, namely ACINUS, ATF7IP, CDYL, DNMT1, EHMT1, EHMT2, HDAC1, LIG1, NUP107, and WIZ [63,70,79]. All tested novel candidates demonstrated binding energies within the range of previously identified substrates, supporting their feasibility as non-histone methylation targets (Figure 5D). Indeed, two novel candidates, UHRF1 and PAF1, have higher binding energies than each of the 10 known non-histone substrates of EHMT2. For novel candidates with calculated lower energies, such as POLR1A and CDC73, their measurements were comparable in range to the confirmed EHMT2 substrates ACINUS, CDYL, and WIZ [62]. Furthermore, our results reveal the importance of side chain-mediated interactions for each peptide due to the loss of interaction energy observed with the H3-A^10^ peptide. Thus, if accessible, the histone mimicry motifs present in this novel group of interactors have the potential to bind sufficiently to the EHMT2 substrate peptide-binding groove with similar interaction energies as established substrates of EHMT2. Combined, the current study supports a novel subset of proteins from our interactome dataset as likely non-histone substrates for methylation by EHMT2.

## Discussion

Previous studies from our laboratories have focused on understanding how the histone code functions in human disease, documenting the importance of the H3K9 methylation pathway and its associated epigenetic regulators in monogenic disorders [80,81] and cancer [11,82,83]. Both types of disease processes are believed to originate, at least in part, from alterations in cell cycle progression. Thus, the present study was designed to gain further insight into the mechanisms used by epigenomic regulators of the H3K9me pathway during cell cycle progression, using the well-characterized HeLa cell synchronization model [18]. Combined, the results of these analyses demonstrate that EHMT1, EHMT2, CBX3 and CBX5 establish common and distinct interactions during cell cycle progression. Through these experiments, we also expand the repertoire of proteins linked to these epigenetic regulators independent from their histone association. Moreover, we show that these H3K9me writers and readers form extensive connections with protein complexes from different epigenomic pathways. By extending our analyses to H3K9 histone mimicry, we provide evidence of known and novel protein networks that are candidates for another layer of regulation through methylation by the studied writers. This network of proteins supports additional functional inferences of both mechanistic and biomedical importance. Consequently, in light of this novel information, it becomes pertinent to discuss the mechanistic importance of our findings as well as the cell biological and biomedical relevance to monogenic diseases and cancer.

Cell cycle progression contributes to pathobiological mechanisms underlying Kleefstra syndrome, which is a congenital disease caused by *EHMT1* mutations. Like many monogenic diseases with developmental delay, it is believed that alterations in the asymmetric cell division and migration of neuroblasts play a role in disease presentation [84]. Alterations in nuclear functions are expected to increase the fragility of neuronal nuclei, leading to a reduction in numbers or differentiation of cells, caused by aberrant asymmetric cell division. Notably, the contributions from alterations in this pathway to cancer are more substantiated [85–89]. For example, recent studies demonstrate that H3K9me2 levels are altered in pancreatic cancer [90]. In addition, we have shown that pharmacological inactivation of both EHMT1 and EHMT2 alters pancreatic cancer cell growth [7]. Moreover, we find that this pharmacological inactivation can synergize with the ATR-CHK1 pathway, which is the main signal for replication stress. Congruently, this EHMT-mediated pathway has also been associated with DNA replication, which signals through ATR/ATM [91]. Thus, the evidence reported here underscores the biomedical importance of advancing our understanding of cell cycle-mediated events.

Considering these four H3K9me regulators at the level of systems biology, we have considered its emergent properties outside the canonical H3K9 methylation pathway. First, we find that while the EHMT1 and EHMT2 writers do not directly interact with the CBX3 and CBX5 readers in G1/S, intriguingly, these regulatory systems organize as two distinct reader–writer pairs, namely EHMT1–CBX5 and EHMT2–CBX3 through their shared interactomes. The observed organization of overlapping interactomes between these reader-writer pairs has functional implications for their roles in distinct processes in this cell cycle phase, as the cell either prepares or proceeds to copy its DNA. For example, EHMT2 and CBX3 exhibited associations with proteins relevant to replication fork biology and regulation of DNA methylation through interactions with DNMT1, UHRF1, and USP7, facilitated by histone mimicry mechanisms [54]. Alternatively, EHMT1 and CBX5 displayed interactions with proteins implicated in DNA damage pathways, including ATR, ATRIP, MTA1, and BCLAF1 [52,53]. As the cell progressed to the G2/M phase, EHMT1 maintained these interactions, while CBX5 did not. Remarkably, most of the observed proteins exhibited histone-mimicry-like motifs, suggesting lysine methylation as a potential regulatory mechanism in these protein interactions. While interactions of the H3K9me writers and readers with some of these proteins have been previously shown [15,70,72], their temporal association during the cell cycle and preference with specific writer-reader pairs are novel aspects of the present study that provide a contextual framework to better understand how this epigenomic pathway functions. Evaluating the emergent properties from the system displayed at the G2/M phase, we observe that all four proteins form a quantitatively larger overlapping interaction network compared to G1/S, and the CBX3 and CBX5 interactomes include members of the EHMT writer complex. Notably, this G2/M overlap is largely captured by CBX5 rather than the selective writer-reader pairs driving the shared G1/S networks. Gene ontology analyses of these interactions uncover the enrichment of functions associated with helicase activity, chromatin remodeling, and transcription factor binding. Therefore, these findings support the notion that H3K9me reader proteins participate independently in centromeric and pericentromeric chromatin remodeling with transcription factor bookmarking during G2/M phase. These novel insights into preferential cell cycle mechanisms contribute to a deeper understanding of the complex interplay between epigenetic regulators and cellular processes, highlighting their potential significance in various biological contexts.

An important emergent property of the identified interactomes, independent of the cell cycle, is its link with complexes that regulate other pathways important for the epigenome. For instance, EHMT2 interacts with the ALL-1 Supercomplex (ALL-1), which forms a membrane-less nuclear aggregation of more than 20 proteins that are part of the Sin3-HDAC, the NuRD histone deacetylase (Nucleosome remodeling and deacetylation), and SWI/SNF (SWItch/Sucrose Non-Fermentable) protein complexes [92]. This not only provides further insight into the extensive interaction network among these epigenomic protein complexes, but intriguingly, these protein complexes also contain candidates for histone mimicry by these KMTs. The findings that interactions with these pathways are not cell cycle dependent and include several targets for H3K9 mimicry support the importance of cross-epigenomic pathway interactions to maintain basic cell functions. Moreover, this interconnectedness with other types of epigenomic complexes reinforces the need for broader considerations when pharmacologically targeting not only EHMT1 and EHMT2 but other epigenomic regulators as well.

In summary, the present study is the first comprehensive characterization of the repertoire of proteins that interact with the H3K9me writers (EHMT1 and EHMT2) and readers (CBX3 and CBX5), which reveal systems-level organization across cell cycle phases. We deconvoluted the cellular interactomes into cytoplasmic and nuclear proteins to develop specific G1/S and G2/M pathways and networks. In addition to examining these protein–protein interactions, we investigated the extensive repertoire of proteins that can be potentially methylated and/or read by these writers and readers. While the H3K9me mark is consistently present in both G1/S and G2/M [93], the emergent properties from the current study indicate that functional roles of its writers and readers vary across the cell cycle to differently regulate linked cellular biology programs. This new knowledge is not only important for better understanding the context in which these epigenomic regulators operate but also for the potential implications of targeting these pathways, through EHMT1/EHMT2 inhibitors, in combination with G1/S phase inhibitors (e.g. cdk4/cdk6) or those for G2/M (e.g. Aurora A). Thus, we are optimistic that our results will inform aspects of both, cancer pathobiology and therapeutics.

## Supplementary Material

Supplementary Figure S1

Supplementary Tables S1-S4 and Supplementary Data S1

## Data Availability

All proteomic datasets are provided in Supplementary Materials. MD Simulation data will be available upon request.

## Abbreviations

ALL-1: ALL-1 Supercomplex
CBX: chromobox
G1/S: GAP 1/Synthesis
G2/M: GAP 2/Mitosis
GFA: Genetic Function Approximation
HBD: hydrogen bond donor
HP1: heterochromatin protein 1
KMT: lysine methyltransferase
LC/ESI-MS/MS: Liquid Chromatography Electrospray Ionization Tandem Mass Spectrometry
MD: molecular dynamics
MEME: Multiple Em for Motif Elicitation
NuRD: nucleosome remodeling and deacetylation
PDB: Protein Database
PI: positive ionizable
SAM: S-adenosylmethionine
Sin3A: SIN3 transcription regulator homolog A
SLiM: short linear motif
SMCC: SRB- and MED-containing cofactor complex
SWI/SNF: SWItch/Sucrose Non-Fermentable
TFIIH: transcription factor IIH
