# Supplementary material for: Writers and readers of H3K9me2 form distinct protein networks during the cell cycle that include candidates for H3K9 mimicry
Source: Biosci Rep. 2023 Oct 27;43(10):BSR20231093. doi: 10.1042/BSR20231093 (PMC10611923; doi:10.1042/BSR20231093)
Supplement: Supplementary Figure S1 [file BSR-2023-1093_supp.pdf]

Supplementary figure 1

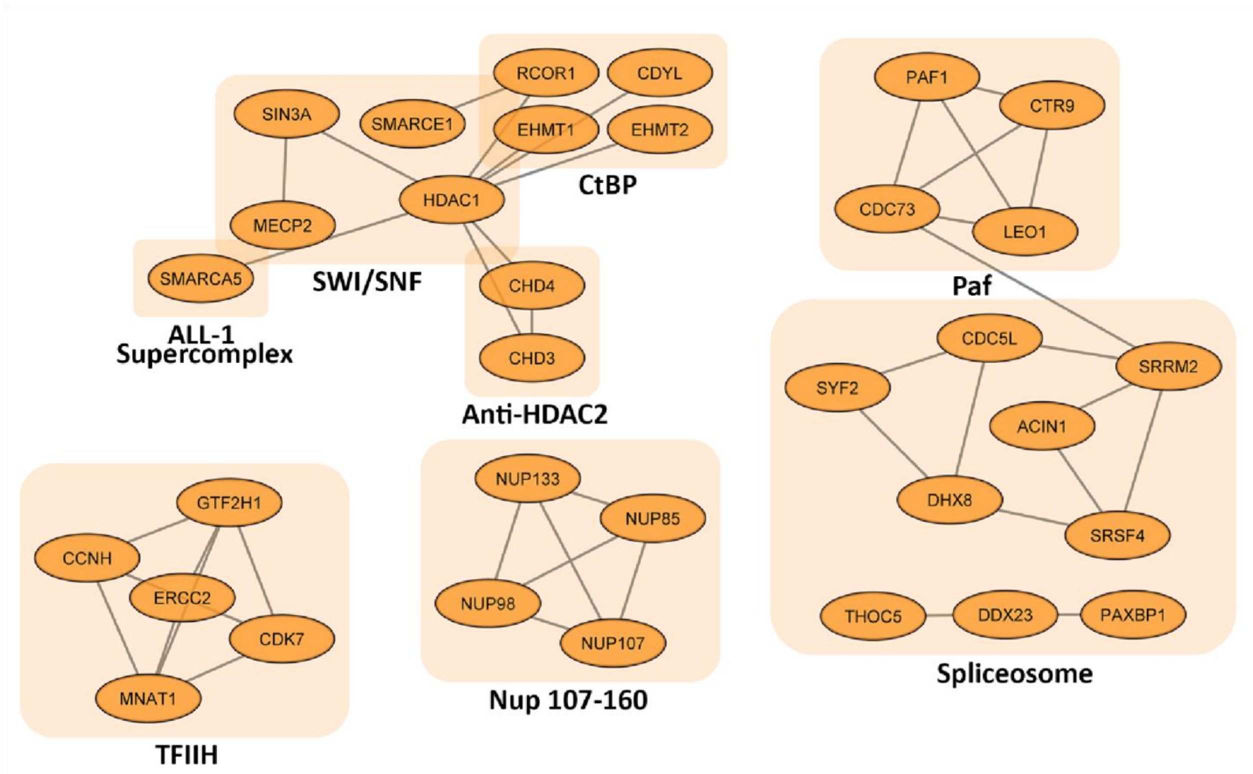

Figure S1: Protein complexes formed by candidates of histone mimicry. CORUM enrichment of interactors demonstrates common core complexes emerging from the histone mimicry candidates.
